# The emergence and outbreak of multidrug-resistant typhoid fever in China

**DOI:** 10.1038/emi.2016.62

**Published:** 2016-06-22

**Authors:** Meiying Yan, Xinlan Li, Qiaohong Liao, Fang Li, Jing Zhang, Biao Kan

**Affiliations:** 1 Department of Diarrheal Disease, State Key Laboratory for Infectious Disease Prevention and Control, National Institute for Communicable Disease Control and Prevention, Chinese Center for Disease Control and Prevention, Beijing 102206, China; 2 Department of Diarrheal Disease, Collaborative Innovation Center for Diagnosis and Treatment of Infectious Diseases, Hangzhou, Zhejiang 310000, China; 3 Department of Diarrheal Disease, Xinjiang Center for Disease Control and Prevention, Urumqi, Xinjiang 830000, China; 4 Department of Diarrheal Disease, Chinese Center for Disease Control and Prevention, Beijing 102206, China

**Keywords:** multidrug resistance, outbreak, typhoid fever

## Abstract

Typhoid fever remains a severe public health problem in developing countries. The emergence of resistant typhoid, particularly multidrug-resistant typhoid infections, highlights the necessity of monitoring the resistance characteristics of this invasive pathogen. In this study, we report a typhoid fever outbreak caused by multidrug-resistant *Salmonella enterica* serovar Typhi strains with an ACSSxtT pattern. Resistance genes conferring these phenotypes were harbored by a large conjugative plasmid, which increases the threat of *Salmonella* Typhi and thus requires close surveillance for dissemination of strains containing such genes.

## Introduction

Typhoid fever, which is caused by *Salmonella enterica* serovar Typhi, remains a major public health threat in developing countries. Approximately 13.5 million cases occur annually, and the disease was associated with 190 000 deaths worldwide in 2010.^1^ In developed areas, such as Europe and North America, the incidence of typhoid fever is very low, whereas in many developing countries, such as Africa, Latin America and South or Southeast Asia, the disease is endemic. For example, the annual incidence of typhoid fever has been as high as 573 cases per 100 000 individuals in Indonesia.^2, 3^

Since the late 1980s, multidrug-resistant (MDR) strains of *S. typhi* that exhibit resistance to chloramphenicol, ampicillin and trimethoprim have increased and spread in Southeast Asia, Central Asia, South America and Africa.^4, 5, 6, 7, 8^ In recent decades, ~10 000–20 000 typhoid cases have been reported annually in China, and outbreaks are not uncommon;^9^ however, MDR *S. typhi* strains were not been detected in China before October 2010, when MDR Typhi strains with resistance to ampicillin, chloramphenicol, streptomycin, trimethoprim/sulfamethoxazole and tetracycline (ACSSxtT) emerged and caused an outbreak in Shache county in the Xinjiang province.

## Materials and Methods

### Ethics statement

This study was reviewed and approved by the ethics committee of the National Institute for Communicable Disease Control and Prevention, China CDC, according to the medical research regulations of the Ministry of Health, China (ICDC-2014008).

### Epidemiological investigation

The epidemiological investigation was conducted as previously described,^10^ with some improvements. In brief, a clinical diagnosis of typhoid fever was defined as a persistent fever (⩾38 °C for >three days) accompanied by headache, hypoeosinophilia or a positive Widal test in endemic regions and without obvious upper respiratory or urinary tract infections, trauma or other diagnosed causes of fever. A laboratory-confirmed case was one with a positive culture from blood or stool. A case–control study with 2:1 matching of controls to cases was initiated after an individual epidemiological case study had been conducted. Controls were healthy individuals who had not had a fever in the month before the study; the samples were matched for age (no more than five years between cases and controls), gender and residential location (controls were enrolled from households adjacent to those of cases). Over a 1-month period, epidemiological experts conducted face-to-face interviews using standard questionnaires to obtain information on exposure history. The solicited exposure history focused on close contact with typhoid cases, travel history, and food and water consumption. Every control and matched case study subject was asked about the same time period of exposure history. All epidemiological data were collected and analyzed using SPSS 17.1 software (SPSS Inc., Chicago, IL, USA). To identify correlations between exposure factors and illness, the odds ratios and exact 95% confidence intervals were calculated via a matched multivariate analysis through conditional logistic regression.

### Laboratory detection

During the outbreak period, blood and stool samples were collected for the suspected cases and cultured according to a previously described method.^11^ The resultant suspected *S*. *typhi* colonies were identified using biochemical tests (Microbact, Medvet Diagnostics, Adelaide, Australia) and serological agglutination reaction. Antimicrobial susceptibility testing was performed using the broth microdilution method according to the instructions of the Clinical Laboratory Standards Institute (CLSI).^12^ Antimicrobial agents, including ampicillin, amoxicillin/clavulanic acid, ceftriaxone, cefotaxime, ceftazidime, nalidixic acid, ciprofloxacin, chloramphenicol, gentamicin, kanamycin, streptomycin, sulfisoxazole, trimethoprim/sulfamethoxazole, tetracycline and azithromycin, were tested and interpreted according to the CLSI minimum inhibitory concentration (MIC) interpretive standards for Enterobacteriaceae to evaluate the resistance levels.^13^ Quality control was performed with the standard strain *Escherichia coli* ATCC 25922 according to the CLSI guidelines.

### Transferability of plasmid by conjugation

To determine the transferability of the MDR plasmid, wild-type *S*. *typhi* strains were tested as donor strains with *E. coli* K-12 MG1655 as the recipient strain in conjugation experiments.^14^ Transconjugants were selected as lactose-fermenting colonies on MacConkey agar plates containing ampicillin and chloramphenicol (50 μg/mL each). The resultant *E. coli* transconjugants exhibited the same resistant profiles as those of the wild-type *S*. *typhi* strain. The conjugation rates of the plasmid were calculated as the number of resultant *E. coli* transconjugants divided by the number of wild-type *E. coli* strains.

### Identification of antimicrobial resistance gene markers by PCR

The resistance genes, including *cat*, *tetA*, *bla*, *strA-strB*, *dfrA* and *sul1*, which are responsible for the resistance of chloramphenicol, tetracycline, ampicillin, streptomycin, trimethoprim and sulfonamide, respectively, were amplified by PCR using previously published primers,^15^ and the PCR products were sequenced by TaKaRa Biotech Co., Ltd (Daliang, China). The target gene was determined by comparing the sequence data with the National Center for Biotechnology Information database sequences using the BLAST program. The quinolone resistance determinant regions (QRDRs) of the DNA gyrase *gyrA* and *gyrB* genes and the DNA topoisomerase IV *parC* and *parE* genes were obtained by PCR, and the amplicons were sequenced to determine single-nucleotide polymorphisms (SNPs) in comparison with *S. typhi* CT18.

### Pulsed-field gel electrophoresis analysis

Pulsed-field gel electrophoresis (PFGE) was performed according to the international PulseNet protocol for *Salmonella* using a CHEF DRIII system (Bio-Rad, Hercules, CA, USA). Band similarities were analyzed using BioNumerics software (version 2.5, Applied Maths, Kortrjk, Belgium) and the unweighted pair-group method with arithmetic means clustering to produce a dendrogram with 1.5% position tolerance. The outbreak strains of *S*. *typhi* from Shache county were subjected to PFGE analysis and molecular comparison.

To detect and determine the size of the large plasmids harbored by the MDR strains of *S. typhi*, S1-PFGE was performed as previously described.^16^ After lysis of bacterial cells embedded in agarose, plugs were digested with S1 nuclease to convert the plasmids into linear molecules and subjected to PFGE. The sizes of the large plasmids were estimated by comparing them with DNA markers.

### Multilocus sequence typing analysis

Multilocus sequence typing (MLST) was performed as described on the MLST website (MLST database for *S. enterica*; http://mlst.ucc.ie/mlst/dbs/Senterica). In brief, for each isolate, seven housekeeping genes—*hisD*, *aroC*, *dnaN*, *hemD*, *purE*, *sucA* and *thrA*—were amplified by PCR using the primers on the MLST website. Amplicons were sequenced by TaKaRa Biotech Co., Ltd. MLST allele numbers and sequence types were determined by submitting the sequence data to the MLST database of *S. enterica* and then running a search. MLST typing of the plasmids (PMLST) harbored by the MDR isolates was performed as previously described.^17^ The PMLST alleles and sequence types of the plasmid were identified and designated by comparing the database of PMLST using the DNA sequences of six conserved genes on the plasmid.

### Whole-genome sequencing, comparison and phylogenetic analysis of *S. typhi* isolates

Whole-genome sequencing (WGS) was performed on one representative outbreak strain and four endemic strains circulating in other provinces before the outbreak in China using an Illumina HiSeq 2000 (Illumina, San Diego, CA, USA) with 500-bp paired-end libraries in eightfold multiplexes (Beijing Genome Institute, Shenzhen, China). Genomic DNA of *S. typhi* was prepared using a Wizard Genomic DNA Purification Kit (Promega, Madison, WI, USA) according to the manufacturer's protocol. The sequence assembling, alignment and quality control were performed as previously described.^11^ The accession number of the five strains was PRJEB7510 (Table 1). By comparing the genomes of the five strains and 13 published *S. typhi* genomes in GenBank (Table 1), we identified 2803 SNPs after removing SNPs in recombinant regions, as suggested by ClonalFrame.^18^ A maximum likelihood tree was inferred from the identified 2803 SNPs using MEGA v5.^19^ The genomic sequence of E02_1180 was determined as the root because it was most distantly related to the outbreak isolates.

## Results

### Typhoid fever epidemic in Shache

The first case in this outbreak was reported on 10 July 2010, in Shache county. By the end of the outbreak in December 2010, 253 cases (55 laboratory-confirmed and 198 clinically diagnosed cases) were reported from 26 towns in Shache.^10^ Eighty-four percent of the patients were farmers and students between seven and 45 years of age. The male-to-female ratio was 1.2:1. Most cases (185, 73.1%) were reported from the town of Ailixihu, which was inconsistent with the low number of cases reported in this town in the five years before the outbreak (an average of four sporadic typhoid fever cases each year from 2005 to 2009). As a result of the outbreak, the incidence of typhoid fever in Ailixihu reached 265.8/100 000 in 2010, whereas the national incidence was 1.05/100 000. As the outbreak subsided, the reported cases decreased markedly after November throughout all of Shache county.

### Laboratory investigation

**Figure 1:**
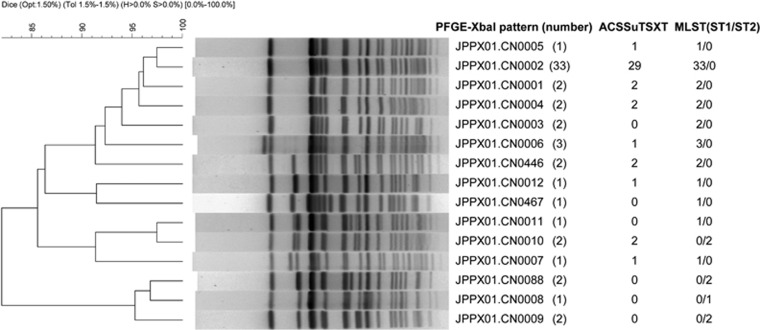
Molecular types and antimicrobial phenotypes of *S*. *typhi* isolates.

During the outbreak, 55 *S. typhi* strains were obtained from the blood or stool of 55 patients. One isolate was recovered from a suspected source of drinking water. All 56 isolates were analyzed by PFGE with *Xba*I digestion. Thirteen patterns were identified (Figure 1). Thirty-three (59% of 56) isolates clustered to the dominant pattern JPPX01.CN0002. This pattern matched six strains that were isolated from 2001 to 2004 in other counties of Xinjiang but did not match any *S. typhi* isolates collected from other provinces in China. Antimicrobial susceptibility testing indicated that most *S. typhi* isolates in this outbreak (41/56) were MDR and contained the same resistance (R)-type ACSSxtT pattern. Twenty-nine of the 41 (R)-type ACSSxtT strains, including the strain isolated from drinking water, belonged to the dominant pattern JPPX01.CN0002. The historical 6 JPPX01.CN0002 strains isolated from 2001 to 2004 in Xinjiang were susceptible to ACSSxtT, suggesting that they may be the evolutionary progenitor of ACSSxtT-resistant strains. In addition, six outbreak isolates (four of which were ACSSxtT-resistant) exhibited resistance to nalidixic acid (MIC>128 mg/mL) and decreased susceptibility to ciprofloxacin (MIC=0.125–0.5 mg/mL). A single *gyrA* (S83F/D87N) mutation was identified via PCR upon screening of the QRDRs of *gyrA*, *gyrB*, *parC* and *parE* genes in these strains. All hospitalized patients were administered fluoroquinolones, including oral norfloxacin (0.2 g, three times daily) or intravenous levofloxacin (0.2 g, once daily) combined with ceftriaxone (2.0 g, intravenously, twice daily), for 2–3 weeks. This antibiotic therapy was effective for most of the patients.

**Figure 2:**
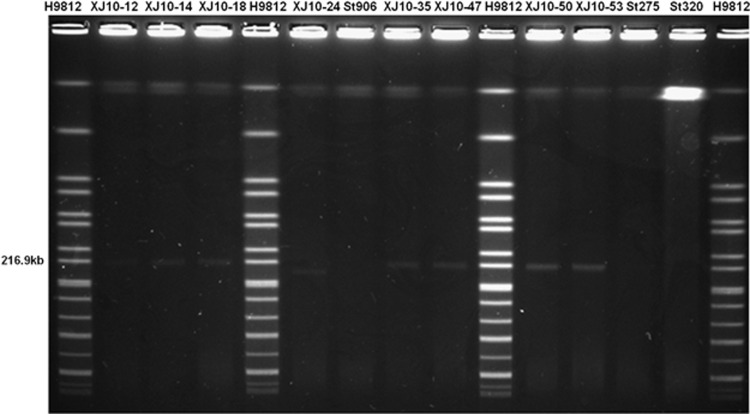
Mobility of large plasmids in pulse-field gels after S1 digestion. The sources of DNA were as follows: markers H9812 digested with *Xba*I (lanes 1, 5, 10 and 15); outbreak strains (lanes 2–4, 6, 8–9 and 11–12); and pan-susceptible pre-outbreak strains (lanes 7 and 13–14).

An S1-PFGE analysis of the ACSSxtT strains in this outbreak and some historical circulating strains before this outbreak showed that all outbreak MDR isolates harbored a plasmid of ~217 kb (Figure 2), which was similar to the size of the plasmid pHCM1 (218 160 bp) in *S*. *typhi* CT18. Plasmid pHCM1 is known to carry multi-resistance genes. One outbreak strain resistant to chloramphenicol and tetracycline had a plasmid of ~180 kb, yet no plasmid was found in the pre-outbreak isolates in this region (Figure 2).

To determine the transferability of this ∼217-kb plasmid, a conjugation experiment was performed with the wild-type ACSSxtT strains as donors and *E. coli* MG1655 as the recipient strain. The conjugation rates of this plasmid ranged from 1.5 × 10^−3^ to 6 × 10^−4^ using ten randomly selected ACSSxtT outbreak strains, indicating that the ACSSxtT plasmid was transferable. However, the 180-kb plasmid in the *S*. *typhi* outbreak strain could not be transferred to *E. coli* MG1655.

### Molecular analysis of the outbreak strain

MLST of 56 outbreak strains revealed a predominant ST1 (49/56) subtype and a minor ST2 (7/56) subtype (Figure 1). All outbreak strains belonged to the *S. typhi* H58 haplotype by PCR amplification and sequencing. The analysis of the MDR plasmid type from 15 randomly selected ACSSxtT-resistant strains showed that each carried plasmid pSTY7, which was first reported in 1993. The MLST of the plasmid showed that it belonged to the sequence-type ST6.

In addition, we selected one outbreak strain (XJ10–14) with the dominant PFGE pattern JPPX01.CN0002 and four endemic strains from four other provinces with a high incidence of typhoid fever for WGS. On the basis of the genome comparison and PCR amplicon sequencing, resistance genes were found in the plasmid segments, including *cat*, *tetA*, *bla*, *strA*-*strB*, *dfrA* and *sul1*, which are responsible for resistance to chloramphenicol, tetracycline, ampicillin, streptomycin, trimethoprim and sulfonamide, respectively. Combining the results of genome sequencing and transfer of the resistant markers, we further confirmed that the resistance genes were located on the transferable ∼217-kb plasmid.

**Figure 3:**
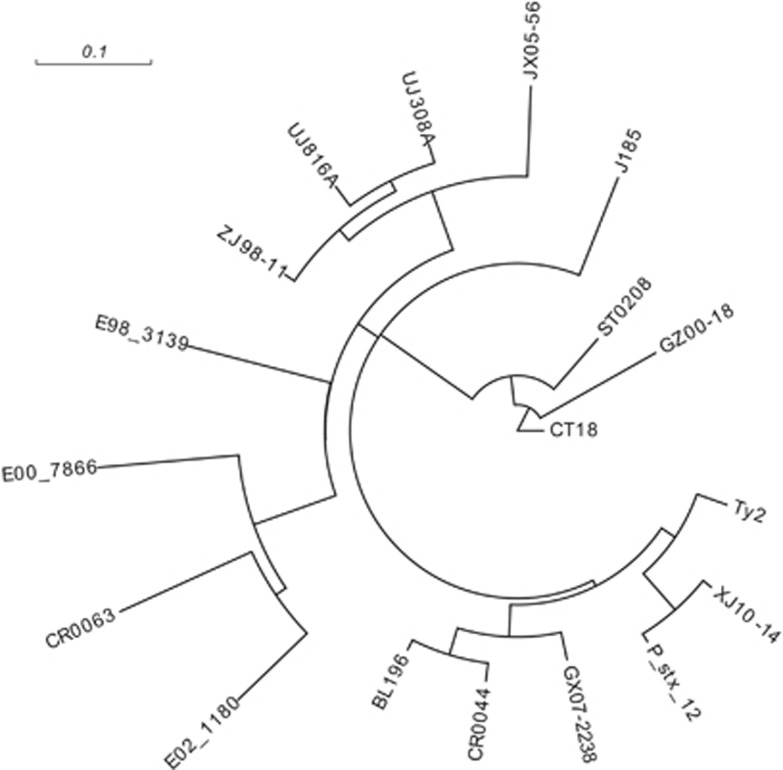
Maximum likelihood tree of 18 *S*. *typhi* isolates based on an SNP analysis. In the tree, the strain E02_1180, representing haplotype H45, serves as the root.

The sequenced genome of the outbreak strain XJ10–14 was compared with four other sequenced genomes of Chinese *S. typhi* strains along with additional genomes obtained from GenBank (Table 1). A maximum likelihood tree (Figure 3) was inferred from the identified 2803 SNPs. A close relationship was discovered between XJ10–14 and a chronic carrier strain, P_stx_12, isolated in India, whereas four strains from different provinces of China belonged to different branches of the tree, which indicated that the Shache outbreak strains were most closely related to the Indian carrier strain. They had higher genomic similarities compared with the strains from other regions of China; thus, the outbreak strain potentially resulted from the transmission of a clone containing P_stx_12 from India. Using another strain, E02_1180, which was also from India and was far away from the above two strains in the likelihood tree, we could not rule out the possibility that several clones of *S. typhi* were circulating in India,^15^ one of which was transmitted into China by human travel. Three other *S. typhi* strains, JX05–56, ZJ98–11 and GX07–2238, from southeastern China were clustered together with four strains (UJ308A, UJ816A, BL196 and CR0044) from Malaysia, indicating that the typhoid fever that occurred in China was related to that in Malaysia.

In addition, we analyzed the genomes of a carrier group (three isolates from two countries) and a patient group (15 isolates from eight countries). In total, 1422 SNPs were found in the carrier group. Within these SNPs, 464 were synonymous and 389 were nonsynonymous. In the patient group, 2508 SNPs were observed, with 389 synonymous and 916 nonsynonymous mutations. There were 2803 different SNPs between these two groups, involving 1278 genes annotated in the reference stain CT18. We also identified 1281 and 66 specific genes in the carrier and patient groups, respectively. The identified mutations (SNPs) in the core genome and specific genes in the pan-genome between the two groups revealed the genome evolution and mechanism involved in carrier-state transformation. Because these strains were obtained from different events (e.g., different years, locations and sources), no high similarities or epidemiological correlation was observed between the carrier individuals and the patient individuals.

We also analyzed the sequences of XJ10–14 (an MDR strain) and Ty2 (a non-resistant strain). A phylogenetic relationship was observed between these two strains. Ty2 was an assumed ancestor of XJ10–14 owing to its evolutionary position based on the 131 identified SNPs. Of these SNPs, 108 were located in 91 ORFs, causing 26 amino-acid mutations (nonsynonymous) in 26 proteins. According to the phylogenetic tree and the whole-genome data, XJ10–14 probably evolved from Ty2-like strains through the accumulation of mutations and MDR plasmid acquisition.

A comparative genomic analysis of the MDR plasmids from XJ10–14 and CT18 showed that the pHCM1 plasmid from CT18 contained 239 genes, and 207 homologs of these genes were found in the MDR plasmid from XJ10–14 strain. Furthermore, 94 of the 207 genes displayed 100% identity of nucleotide sequence, whereas the others exhibited >77.9% identity. These results indicate that the MDR plasmid in the XJ10–14 strain may have originated from pHCM1-like divergent plasmids in *S. typhi*.

The alignment of resistance genes conferring the ACSSuTSXT-resistant phenotype on MDR plasmids from XJ10–14 and CT18 (these two strains shared the same resistance genes) and the corresponding genes in SGI-1 islands from nontyphoidal *Salmonella* showed high diversity. The *tetA* gene was observed on MDR plasmids in *S. typhi* strains, whereas the *tet(G)* gene was conserved in SGI-1 islands. The sequences of *floR* (conferring resistance to chloramphenicol/florfenicol) and *tet(G)* genes are similar among nontyphoidal *Salmonella*, whereas *cat* genes (responsible for chloramphenicol resistance) were preserved in *S. typhi*. A similar situation was observed in streptomycin resistance genes, with *aadA2* in SGI-1 islands and *strA-strB* on MDR plasmids. Moreover, the gene organization of antimicrobial resistance genes in SGI-1 islands was different from those on MDR plasmids. All five resistance genes were found in a 13-kb multidrug resistance region consisting of an unusual class I integron structure related to In4 in SGI-1 islands, whereas the five genes on MDR plasmids of *S. typhi* seemed to be distributed independently.

### Intervention during the outbreak

Epidemiological studies revealed that the consumption of raw tap water from the main water tower of the town and cold rice foods from street stalls in the Bazha area were closely associated with the outbreak.^10^ Intervention measures taken in Ailixihu, including rectifying the sanitation conditions of the street stalls, building a new water tower, disinfecting the water, enacting health education, and administering the Vi vaccine, led to an end of the outbreak in November 2010.

## Discussion

*S. typhi* with multidrug resistance to first-line antimicrobials has been emerging worldwide.^20, 21, 22^ In this study, we identified the outbreak strains with an MDR pattern of ACSSxtT, and the resistance genes conferring these phenotypes were harbored by a large conjugative plasmid. Not only is this the first time that this phenotype has caused an outbreak in China; it is also the first time it has been observed in China. A similar pattern was reported in sporadic cases in China, but either the isolates displayed different PFGE patterns^23^ or the resistance genes were located either in the integron I island on the chromosome or on an IncC plasmid.^24^ Other multidrug resistance isolates with different resistant profiles were found in other areas of China.

The emergence of MDR *S. typhi* results in the excessive use of ciprofloxacin for typhoid fever treatment. In the 1980s, ciprofloxacin became the first-line drug to treat *S. typhi* infections after most of the conventional drugs became ineffective.^25^ However, resistance to ciprofloxacin was observed in the early 1990s,^26^ and treatment failure with this antibiotic was reported by the late 1990s.^27^ Several outbreaks associated with nalidixic acid-resistant *S. typhi* that showed a decreased susceptibility to fluoroquinolones occurred.^28^ In our study, specific outbreak strains also showed decreased susceptibility to fluoroquinolones, which was induced by mutation of the *gyrA* gene. Taken together, these results indicate the global spread of multidrug resistance and decreased ciprofloxacin susceptibility in *S. typhi* clones.

As a result of the emergence of MDR and quinolone-resistant isolates, alternative antimicrobial treatment regimens, such as the use of ceftriaxone and azithromycin, have been recommended to clinicians for *S. typhi* infections.^5, 21, 29, 30^ No azithromycin-resistant isolates have been found in China, but resistance to this antibiotic has recently been observed elsewhere.^31, 32^ The development of antimicrobial resistance calls for restrictive use of antimicrobials and the establishment of a nationwide and global real-time antimicrobial surveillance network to monitor the development of antimicrobial resistance and initiate intervention at an early stage.

All the studied outbreak strains belonged to the *S. typhi* H58 haplotype, which is widespread in Southeast Asia,^33^ indicating the global spread and clonal expansion of this type of strain. However, it is unclear whether the (R)-type ACSSxtT isolates circulating in Southeast Asia^17, 34, 35^ are associated with the outbreak strain in China because of the lack of detailed historical epidemiological data and comparative molecular typing information for the *S. typhi* isolates in these regions.^17, 34, 36^ The close relationship between the outbreak strain and the Indian strain indicates a potential origin and transmission of the outbreak strain from India. The increase in travel to India, particularly for worship or religious reasons, may have led to the introduction of *S. typhi* H58 into western China, including Xinjiang Province. A more plausible hypothesis is that an ancestral *S. typhi* H58 strain spread from India to Xinjiang, where it evolved and acquired the MDR islands on the IncHI1 plasmid. To test this hypothesis, historical and contemporary isolates from China, India and Southeast Asia, specifically Vietnam, need to be further investigated.

In summary, the 2010 Shache outbreak is the first reported outbreak caused by MDR *S. typhi* with an uncommon MDR pattern of ACSSxtT in an endemic province in China. Whole-genome comparison and drug resistance analysis indicated that the Shache typhoid outbreak was most likely related to endemic MDR strains in Southeast Asia but not eastern China, which suggests an introduction of such strains into western China followed by its expansion in a district with inadequate sanitation. Importantly, the decreased susceptibility to fluoroquinolones of some of the isolated ACSSxtT strains may be the cause of treatment failure, prolonged hospitalization and extensive transmission of the disease. Therefore, it is necessary to track the source of the MDR strains and plasmids responsible for the MDR phenotypes and to monitor their spread through laboratory and extensive epidemiological investigations.

## Figures and Tables

**Table 1:** *S*. *typhi* isolates revealed using whole-genome sequencing

**Strain**	**Isolation year**	**Source**	**Location**	**NGS project/Refseq**
XJ10–14	2010	Patient	China	PRJEB7510
GZ00–18	2000	Patient	China	PRJEB7510
JX05–56	2005	Patient	China	PRJEB7510
ZJ98–11	1998	Patient	China	PRJEB7510
GX07–2238	2007	Patient	China	PRJEB7510
BL196	2005	Patient	Malaysia	NZ_AJGK00000000.1
CR0044	2007	Carrier	Malaysia	NZ_AKZO00000000.1
CR0063	2007	Carrier	Malaysia	AKIC00000000.1
E00_7866	2000	Patient	Morocco	NZ_CAAR00000000.1
E02_1180	2002	Patient	India	NZ_CAAT00000000.1
E98_3139	1998	Patient	Mexico	NZ_CAAZ00000000.1
J185	1985	Patient	Indonesia	NZ_CAAW00000000.1
ST0208	2008	Patient	Malaysia	NZ_AJXA00000000.1
UJ308A	1998	Patient	Malaysia	NZ_AJTD00000000.1
UJ816A	1998	Patient	Malaysia	NZ_AJTE00000000.1
CT18	1993	Patient	Vietnam	NC_003198.1
P_stx_12	Undefined	Carrier	India	NC_016832.1
Ty2	1916	Patient	Russia	NC_004631.1
